# Comparison of absorbed irrigation fluid volumes during retrograde intrarenal surgery and percutaneous nephrolithotomy for the treatment of kidney stones larger than 2 cm

**DOI:** 10.1186/s40064-016-3383-y

**Published:** 2016-10-04

**Authors:** Vahit Guzelburc, Mehmet Balasar, Mukaddes Colakogullari, Selcuk Guven, Abdulkadir Kandemir, Ahmet Ozturk, Pelin Karaaslan, Bulent Erkurt, Selami Albayrak

**Affiliations:** 1 Department of Urology, School of Medicine, Medical Faculty of Medipol University, TEM Avrupa Otoyolu Goztepe Cikisi No: 1 Bagcilar, 34214 Istanbul, Turkey; 2 Department of Urology, Meram School of Medicine, Necmettin Erbakan University, Konya, Turkey; 3 Biochemistry Department, School of Medicine, Istanbul Medipol University, Istanbul, Turkey; 4 Department of Anesthesiology, School of Medicine, Medipol University, Istanbul, Turkey

**Keywords:** Renal stone, Flexible ureteroscopy, Percutaneous nephrolithotomy, Retrograde intrarenal surgery, Complication

## Abstract

**Purpose:**

Irrigation-induced increase in intrarenal pressure is of concern because it may cause infection due to increased pyelovenous and pyelolymphatic absorption. This study is the first to compare prospectively the absorbed fluid volumes during percutaneous nephrolithotomy (PCNL) and retrograde intrarenal surgery (RIRS) for stones larger than 2 cm.

**Materials and methods:**

General anesthesia was applied to all patients. Isotonic solution containing 1 % ethanol was used as irrigation fluid. Venous blood ethanol concentration was first measured with the start of irrigation and thereafter every 15 min until the patients left the recovery room. Absorbed fluid volumes were measured using the blood ethanol concentrations. Duration of irrigation, irrigated fluid volume, stone size and grade of hydronephrosis were also recorded.

**Results:**

A total of 60 patients were included the study. Fluid absorption occurred in all patients. Minimum and maximum ranges of fluid absorption were 20–573 mL for RIRS and 13–364 mL for PCNL. The increase in fluid absorbed volume was observed as a result of the given amount of irrigating fluid used in the PCNL group. Also prolongation of operation led to a significant increase in absorption in the PCNL group. Increase in body mass index, stone size, and hydronephrosis did not affect fluid absorption significantly in either of the two operation techniques in correlation analyzes.

**Conclusion:**

Both RIRS and PCNL are conducted under high pressure and can be accompanied potential complications such as SIRS. The fluid absorption confirmed in our study should be taken into consideration during RIRS and PCNL.

## Background

Percutaneous nephrolithotomy (PCNL) is the most preferred technique for the surgical treatment of large kidney stones (Turk et al. 2015). With growing experience, retrograde intrarenal surgery (RIRS) is now being used more effectively for treatment of stones (Grasso et al. 1998). Irrigation-induced increase in intrarenal pressure is of concern because it may cause systemic inflammatory response syndrome (SIRS) due to increased pyelovenous and pyelolymphatic absorption (Mulvaney 1963; Stenberg et al. 1988). A limited number of studies have evaluated the fluid absorption occurring due to use of large amounts of irrigation fluids during PCNL (Kukreja et al. 2002). However, factors affecting absorption with high intrarenal pressure during RIRS are not evaluated. This study is the first to compare prospectively the absorbed fluid volumes during two minimally invasive treatment modalities used for Stones larger than 2 cm.

## Patients and methods

The study was conducted prospectively, with the same number of cases from each of two high-volume centers, one performing PCNL and the other performing RIRS. Patients with kidney stones larger than 2 cm who were admitted to hospital between November 2014 and March 2015 were included in the study. Local ethics committee approved the study, and all patients signed the written informed consent. Pediatric patients, patients with positive urine cultures, patients with a history of ethanol abuse or habitual alcohol intake, and patients with significant cardiovascular, hepatic, renal, or psychiatric disorders or pulmonary disease leading to debilitation were excluded, along with patients who were ASA grade ≥3. Patients who had previous surgery for renal stones or who were stented without having operation were excluded as well.

Stone burden and collecting system anatomy were evaluated using computed tomography scan (CT) in all patients preoperatively. Stone size was calculated by the addition of longest diameters.

After 8 h of fasting, general anesthesia was applied and a basal intravenous infusion of 5–6 mL/kg/h of saline was started to all patients. Isotonic solution containing 1 % ethanol was used as irrigation fluid. Irrigation fluid pressure was set as 60 cm/H_2_O. Hand-pump was also used during irrigation if needed however, because experienced urologists performed the operations, hand-pump use was limited. Absorbed fluid volumes were calculated using blood ethanol concentrations. Blood samples were drawn from patients before starting the operation and then at 15-min intervals. Alcohol concentration in blood was calculated by using Ethanol Gen.2 Kit (Roche Diagnostics, Mannheim, Germany) with an automated analyzer (COBAS Integra, Roche Diagnostics, Mannheim, Germany). Alcohol concentration in whole body blood was calculated by multiplying alcohol concentration (mg/L) with total body blood volume. Total blood volume of each patient was calculated using Nadler’s Formula (for males = 0.3669 × height in m^3^ + 0.03219 × weight in kg + 0.6041; for females = 0.3561 × height in m^3^ + 0.03308 × weight in kg + 0.1833) (Nadler et al. 1962). Alcohol concentration in absorbed irrigation fluid is proportional to total alcohol concentration in whole blood. Irrigated fluid volume and grade of hydronephrosis were also recorded. The stone-free rates were assessed with non-contrast CT 1 month after surgery for PCNL. Imaging was not repeated before the second session in patients with known rest stones. The stone free rates of patients whose operation was terminated due to lack of any rest stones, were assessed with non-contrast CT 1 month after internal stent removal for RIRS.

### RIRS technique

Ureteroscopy was performed using a 7 F semi-rigid ureteroscope to visualize till the proximal ureter. Patients with ureter stones or ureteral stricture/obstruction were excluded along with patients who had fever, pyelonephritis or a draining abscess in renal pelvis. A guidewire was inserted into the renal pelvis and a ureteral access sheath (UAS) (Flexor ureteral access sheath 9.5/11.5 F; Cook Medical, Bloomington, IN, USA) was used as a routine. UAS length was chosen as 35/45/55 cm, depending on the height of the patient, and the UAS tip was placed into the proximal ureter under fluoroscopic guidance. If the UAS could not be advanced through a narrow ureter, a double-j stent was inserted and left in place for a month to wait for dilatation. These patients were also excluded from the study. A 7.5 F flexible ureteroscope (FlexX2, Karl Storz, Tuttlingen, Germany) was used in all patients. Stone fragmentation was done by Holmium YAG laser (Sphinx, Lisa Laser, 30 watts, Katlenburg, Germany) with 272 µm (Flexi Fib, Lisa Laser, Katlenburg, Germany) laser fibers. An energy level of 0.6–10 J and a rate of 6–10 Hz were used. Fragmentation was done until the stone size was reduced to less than 4 mm. If a fragmentation lasted longer than 150 min, it was terminated and postponed to a second session 10 days later. For stone analysis, fragments were collected by a sieve from urine at the post-operative spontaneous micturition. Active retrieval of fragments was not performed for any patient. At the end of the procedure, an internal stent, whose length was determined according to the patient’s height (4.8 F, 22–28 cm), was placed in each patient. Patients’ stents were removed 2–3 weeks later.

### PCNL technique

During rigid cystoscopy (21 F cystoscope, Karl Storz, Tuttlingen, Germany), a 6 F open-ended ureteral catheter (Coloplast, Humlebaek, Denmark) was inserted into patients in the lithotomy position under general anesthesia. Subsequently, patients’ position was changed into prone. Following a mixture of saline and opaque material flush, percutaneous renal access was enabled through a ureteral catheter using an 18 G access needle under C-arm fluoroscopy (Ziehm Vision R, Nurnberg, Germany). Up to 28 F Amplatz dilators (Plasti-med, Istanbul, Turkey) were used. Under fluoroscopic imaging, a 30 F Amplatz sheath (Plasti-med, Istanbul, Turkey) was inserted into the collecting system. Single access was done to all patients. A 26 F nephroscope (26 F nephroscope, Karl Storz, Tuttlingen, Germany) was used during the procedure. Stone fragmentation was performed using a pneumatic lithotripter (Lithopulso Digi, Aymed, Istanbul, Turkey), and fragments were extracted via a grasper (Karl Storz, Tuttlingen, Germany). At the end of the procedure, a nephrostomy tube (Rusch, Karnunting, Malaysia) was inserted in each patient and removed on the first postoperative day.

### Statistical analysis

The Pearson Chi Square test and Fisher’s exact test were used for comparisons of the categorical variables. The Mann–Whitney U test and Wilcoxon Signed rank test were used for the comparison of the two groups. Kruskal–Wallis, One way Anova, and Welch test were used for the comparison of more than two groups. Spearman’s Rho test was used to analyze correlations among the variables.

## Results

After approval by a local ethics committee, 30 patients on whom RIRS was performed in Medipol Medical Faculty and 30 patients on whom PCNL was performed in Meram Medical Faculty were included in the study. No statistically significant difference was found among age, gender, body mass index (BMI) and stone sizes of patients (p > 0.05). Duration of operation was significantly longer in the RIRS group (p = 0.001). Irrigation fluid volume was significantly higher in the PCNL group (p < 0.0001). Fluoroscopy screening time was significantly longer in the PCNL group (p < 0.0001) (Table 1).

**Table 1:** Demographic and clinical variables of patients

	RIRS (30)	PCNL (30)	p value
Mean age	48.6 ± 15	53.2 ± 15	0.25
Sex (male/female)	12/18	14/16	0.79
Mean body mass index (kg/m^2^)	28.8 ± 4.7	28.8 ± 3.8	0.99
Stone size (cm)	3.10 ± 1.5	3.33 ± 1.3	0.37
Mean operation time (min)	107.2 ± 38	78.3 ± 52	0.001
Irrigation fluid volume (mL)	2571 ± 1213	9866 ± 8691	<0.0001
Absorbed fluid amount (mL)	133.1 ± 137	90.9 ± 90	0.25
Fluoroscopy screening time (s)	17.8	194.1	<0.0001
Postop stay (day)	1	3.9	<0.0001

While stone-free rate (SFR) after the first session of RIRS was 50 %, it was 83.3 % in the PCNL group. Statistically significant differences occurred with respect to SFRs between the two operation techniques (p = 0.03). Reoperation was done in cases of residual stones larger than 1 cm. Clinically insignificant residual fragments (≤4 mm) (CIRF) were obtained in all patients of the PCNL group after the second session. CIRF were obtained in 83.3 % of patients after the second, 93.3 % after the third, and 100 % after the fourth session of RIRS.

Fluid absorption occurred in all patients. Minimum and maximum ranges of fluid absorption were 20–573 mL for RIRS and 13–364 mL for PCNL. Although four times more irrigation fluid was used in the PCNL group, no statistically significant difference was observed between operation technique and fluid absorption (Table 1). Increase in BMI, stone size, and hydronephrosis did not affect fluid absorption significantly in either of the two operation techniques in correlation analyzes (p > 0.05).

**Fig. 1:**
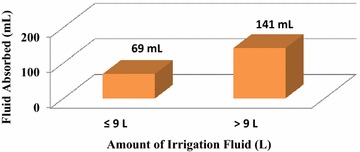
Effect of amount of irrigation fluid used on absorption in PCNL technique

**Fig. 2:**
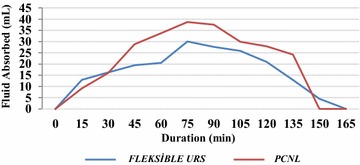
Effect of duration of the operation to fluid absorption in PCNL and RIRS techniques. Only mean instantenous absorption values of ongoing operations were calculated in the graphic. Operations that were terminated were not included in the calculation

The increase in fluid absorbed volume was observed as a result of the given amount of irrigating fluid used in the PCNL group. However, the change in the fluid absorption volume was not observed during RIRS. The volume of fluid absorbed increased with the amount of irrigating fluid used in the PCNL group (p = 0.001), but it was not affected in the RIRS group (p = 0.06). It was noticeable that absorption after 9 L irrigation increased more in the PCNL group (p = 0.005) (Fig. 1). Prolongation of operation led to a significant increase in absorption in the PCNL group (p = 0.0001); however, this difference was not observed in the RIRS group (p = 0.346) (Fig. 2).

No patients developed post-operative electrolyte imbalance. All patients in the RIRS group were discharged on the first post-operative day. Hospital stay (mean 3.9 days) was significantly longer in the PCNL group (p < 0.0001). Blood transfusions were made to two (6.6 %) patients in the PCNL group due to hematuria. No perioperative complication was seen in the RIRS group, and no blood transfusion was made. Pyrexia occurred post-operatively in one (3.3 %) patient in the RIRS group and in five (16.6 %) patients in the PCNL group. Auxiliary procedure was performed on three patients in the RIRS group and two patients in the PCNL group. In the RIRS group, steinstrasse formation was observed in one patient, and the stent was changed. The remarkable thing was that, along with obstruction of the external part of the stent with dust, the interior of the stent was completely obstructed with clay-like dust. During stent removal in the RIRS group, two asymptomatic patients whose stents could not be removed were also seen to have developed steinstrasse formation, and in the same session, the ureter was made stone-free endoscopically. A double-J stent was inserted in one patient due to sepsis and in one patient due to uremia in the PCNL group. One patient developed perirenal hematoma, and spontaneous resolution occurred during follow up.

## Discussion

Systemic fluid absorption was first measured by Hahn et al. during transurethral resection of prostate under spinal anesthesia via addition of ethanol to the irrigation fluid. They measured the absorbed amount by breath ethanol level (Hahn 1988). Because of technical difficulties associated with measurement of breath alcohol concentration under general anesthesia, blood ethanol concentration was directly measured in our study.

Systemic fluid absorption during PCNL can occur through extravasation of fluid due to collecting system perforation, via the vessels that open up during tract dilatation. In addition, absorption can occur due to increased intrapelvic pressure leading to pyelovenous-lymphatic backflow, pyelotubular backflow, and forniceal rupture (Hinman and Redewill 1926; Rao 1987; Matlaga and Lingeman 2012). It has been reported that fluid absorption can occur with intrapelvic pressures as low as 10–20 mmHg (Mulvaney 1963; Stenberg et al. 1988). The amount of fluid systemically absorbed was calculated by using ethanol-containing irrigation fluid, and a value of 178 mL was obtained (Kukreja et al. 2002). Although studies measuring intrapelvic pressure during RIRS and investigating its clinical effects has been done, absorbed fluid amount was not measured in any (Auge et al. 2004; Jung et al. 2008). Factors affecting absorption during both operation techniques have not been evaluated in detail either. Lithotripsy of large kidney stones leads to prolonged operation and increased irrigation fluid amount, both raising concerns about systemic absorption. In this study, we evaluated factors affecting fluid absorption with both operation techniques done for large kidney stones.

A limited number of studies compare SFR of PCNL and RIRS done for large kidney stones. While overall success for RIRS at the end of the procedure was reported as 77–93 %, a meta-analysis showed that PCNL was more successful (Akman et al. 2012; Breda et al. 2008; El-Anany et al. 2001; Hyams and Shah 2009; Mariani 2004; Riley et al. 2009; Mariani 2007; De et al. 2015). In our study, SFR after the first session was significantly lower for the RIRS group. Stone sizes were greater compared to those reported in the literature, because staghorn stones with sizes up to 9.2 cm were included in the study. In addition, we think that SFR is also reduced because only the dusting technique was used for all patients. After a mean of 1.7 interventions, 100 % CIRF was achieved in the RIRS group at the end of the procedure, which is comparable with the PCNL group. Although both operation techniques can be safely used even for large kidney stones, active retrieval of fragments in stones larger than 3 cm will increase SFR of RIRS.

In the only study evaluating fluid absorption during PCNL, Kukreja et al. reported absorbed fluid amounts as 44–474 mL in all patients. It was shown that bleeding during operation, significant perforation of the pelvicaliceal wall, and use of more than nine liters of irrigation fluid increased fluid absorption, although multiple tracts did not affect the amount of absorbed fluid (Kukreja et al. 2002). In our study, absorption occurred in all patients with both minimally invasive operation techniques, regardless of patient and stone characteristics. BMI, grade of pelvicaliceal ectasia, and stone size did not affect absorption amount in either the RIRS or PCNL group. Besides, absorption was not affected by grade of hydronephrosis, pointing out that pyelovenous-lymphatic backflow may not be affected by parenchymal thickness and calyceal anatomy. Increase in the amount of irrigation fluid used and prolongation of operation did not affect fluid absorption in the RIRS group but significantly increased absorption in the PCNL group. In accordance with the previously reported study, we showed that use of >9 L of irrigation fluid was the cut-off value for increased absorption in the PCNL group. Systemic fluid absorption in the RIRS group is thought to be due to increased intrapelvic pressure. Compared to PCNL, RIRS is performed in a relatively closed system, which could be a limiting factor for fluid absorption, leading to mean leakage originating from pyelovenous-lymphatic backflow. Most probably, during PCNL, increased fluid absorption with increasing operation time and irrigation fluid amount after a threshold occurs via the vessels. Questions regarding complicated fluid absorption and the relation between hydronephrosis degree and pyelovenouslymphatic backflow need further research. Additionally, it is remarkable that the amount of irrigation fluid used in the PCNL group for the same stone size is approximately four times higher than in the RIRS group. However there was no significant difference between the two groups with respect to absorption. Relatively decreased absorption with regard to mean fluid usage in PCNL patients could be attributed to the inherent advantage of the 30 F Amplatz sheath. The wider bridge between the collecting system and extracorporeal space allows better drainage.

In the RIRS group, fluid absorption did not increase in relation to any investigated parameter. It should, however, be kept in mind that the amount of absorption can be as high as 573 mL. In the PCNL group, attention should be paid to the fact that prolonged operation time and need of more than nine liters of irrigation fluid can lead to a significant increase in absorption. For both operation techniques, one must be careful about patients with borderline cardiopulmonary or renal status, whose signs of fluid overload can be critical.

Fever after PCNL is a well-documented entity and is reported to be 24–35 % (Rao et al. 1991; Cadeddu et al. 1998; Margel et al. 2006). Another study investigating fever caused by absorption of bacteria and endotoxin-containing fluid reported that increased intrarenal pressure has no relation with post-operative fever (Troxel and Low 2002). In our study fever was found to be more frequent in the PCNL group. We think that pyrexia is increased because of direct fluid absorption via the vessels that open up during renal parenchymal injury. In addition, nephrostomy tubes can facilitate intrarenal colonization during the post-operative period.

There are concerns among urologists that systemic fluid absorption may increase with use of smaller size drainage. While designing our study, we preferred to use the smallest diameter UAS. This made it easier to advance the UAS to proximal ureter in patients with thinner ureters and also enabled us to obtain less irrigation fluid circulation when compared to use of larger diameter UAS. Measurement of absorption even in most unfavorable cases of largest stones, longest operation times and low drainage rate was possible also by the use of small UAS. For a more accurate measurement, the metabolism of alcohol should be considered, too. We ignored the metabolized alcohol amount because of strict exclusion criteria applied and also because of the similarity between mean ages of groups. This may be a limitation of our study. In our study although more absorption occurred with the RIRS technique, fluid absorptions were comparable between the two techniques. The use of single-type UAS and single-size Amplatz sheath is a limitation of our study. Future studies can better explain how intrapelvic pressure affects pyelovenous-lymphatic backflow mechanism by way of evaluating the effect of different size drainage use during RIRS.

## Conclusion

With growing experience, minimally invasive interventions have replaced open surgery. Both RIRS and PCNL are conducted under high pressure and can be accompanied potential complications such as SIRS. The fluid absorption confirmed in our study should be taken into consideration during RIRS and PCNL.

## Abbreviations

PCNL: percutaneous nephrolithotomy
RIRS: retrograde intrarenal surgery
SIRS: systemic inflammatory response syndrome
CT: computed tomography scan
UAS: ureteral access sheath
BMI: body mass index
SFR: stone-free rate
CIRF: clinically insignificant residual fragments
